# How Has It Benefitted the Profession of Texas?

**Published:** 1886-03

**Authors:** 


BRING IT HOME, AND ASK HOW HAS JOURNALISM BENEFITTED THE PROFESSION OF TEXAS.

---

We produce the above excellent article entire, for the benefit of our readers. We endorse every word of it, and we desire to make an application of its truths to the Texas profession. We wish, also, to emphasize the last paragraph, to impress upon the fledgelings, especially, that they do not know what powers they may have, untried; and to start them in the way of their development. First learn to *think out* your subject. *Scribendi recte sapere est et principium et fons*—"the principle and source of good writing, is to think correctly"—observe closely and get into a habit of jotting down your observations.

With reference to the benefits of journalism, we ask our readers to look back a few years, and to contrast the present status of the Texas profession with what it was at the time when there was no medical journal published in Texas. How many local societies have been stimulated into existence? how the State association has developed; how many practitioners who never wrote a paper before. have come into prominent notice through their contributions to the Transactions of the State association, and to the Texas Medical Journal. (See article, "18th annual meeting T. S. M. A.") One article published in this journal (Dr. Wilkinson, Treatment of Carbuncle), has been made the subject of a *leading editorial* in the *Journal of the American Medical Association*; and taken up again, and discussed editorially in terms of commendation in that excellent and able periodical,the *American Practitioner and News.* These articles have circulated and been read all over the world, in Europe and America; while we have had the satisfaction of seeing articles by inexperienced and, heretofore untried writers, in the Transactions, copied far and near.

Verily, the medical journal, properly sustained and managed, is a powerful factor in the development, advancement and progress of the profession; and practically, an educator of physicians. See, too, the fact, a most promising feature of the future of the Texas profession;—every year, Texas students take first honors at the several colleges. Those young men must learn to write for the medical press.

We take this occasion to thank our patrons for the liberal support they have given us in our effort to found a Home journal of a high order. To that support is largely due the brilliant success it has achieved at eight months old. Continue the good work, and the influence of the Journal *for good* will be even more marked than it has been.

